# Secondary outcomes of enhanced cognitive behavioral therapy (eCBT) for children and adolescents with obsessive-compulsive disorder

**DOI:** 10.3389/fnhum.2023.1330435

**Published:** 2024-01-08

**Authors:** Bo Wang, Gudmundur Skarphedinsson, Bernhard Weidle, Lucía Babiano-Espinosa, Lidewij Wolters, Jostein Arntzen, Norbert Skokauskas

**Affiliations:** ^1^Norwegian Centre for E-health Research, University Hospital of North Norway, Tromsø, Norway; ^2^Faculty of Psychology, University of Iceland, Reykjavík, Iceland; ^3^Department of Mental Health, Regional Centre for Child and Youth Mental Health and Child Welfare (RKBU Central Norway), Norwegian University of Science and Technology, Trondheim, Norway; ^4^Department of Child and Adolescent Psychiatry, St. Olav’s University Hospital, Trondheim, Norway; ^5^Accare Child Study Center, Groningen, Netherlands

**Keywords:** cognitive-behavioral therapy (CBT), enhanced cognitive behavioral therapy (eCBT), obsessive-compulsive disorder (OCD), pediatric, children, adolescents, secondary outcomes

## Abstract

**Background:**

Obsessive-compulsive disorder (OCD) is a debilitating mental health condition usually presenting with a high degree of comorbid symptoms in the majority of cases. Although face-to-face cognitive-behavioral therapy (CBT) is considered the therapeutic golden standard for pediatric OCD, its accessibility, availability, and consistency in delivery are still limited. To address some of these challenges, an enhanced CBT (eCBT) package was created and introduced. This study explored eCBT’s broad-based impact on OCD-related comorbid symptoms, functional impairment, quality of life and family accommodation among youth with OCD.

**Methods:**

This open trial involved 25 pediatric patients with OCD (7−17 years), assessed between January 2018 to February 2020. All patients received eCBT for 14 weeks. Secondary outcomes were assessed at baseline, post-treatment, and 3-, 6-, and 12-month follow-up co-occurring symptoms were evaluated using the Strengths and Difficulties Questionnaire (SDQ), Screen for Child Anxiety-Related Emotional Disorders (SCARED), and Mood and Feelings Questionnaire (MFQ). Quality of life was measured using the KINDL-R, functional impairment through the Child Obsessive-Compulsive Impact Scale Revised (COIS-R), and family accommodation by the Family Accommodation Scale (FAS). Linear mixed-effects models were applied to analyze treatment effects.

**Results:**

Results indicated a significant decrease in OCD-related comorbid symptoms post-treatment, with SDQ mean reduce of 3.73 (SE = 1.10, child) and 4.14 (SE = 1.19, parent), SCARED mean reduce of 10.45 (SE = 2.52, child) and 8.40 (SE = 2.82, parent), MFQ mean reduce of 3.23 (SE = 1.11, child) and 2.69 (SE = 1.18, parent). Family accommodation declined with clinician scored FAS mean reduction of 13.25 (SE = 2.31). Quality-of-Life improved significantly post-treatment, with KINDL mean increase of 8.15 (SE = 2.87, children), and 10.54 (SE = 3.07, parents). These positive improvements were further amplified at the 3-month follow-up and remained consistent at the 12-month follow-up.

**Conclusion:**

A significant reduction was observed in all secondary outcomes employed and OCD-related functional impairments from baseline to post-treatment, which was maintained through 12-month follow-up. These results imply that after receiving eCBT, children and adolescents experienced substantial decrease in the negative impacts of OCD-related symptoms on their daily life, including home, school, and social interactions.

## Introduction

Obsessive-compulsive disorder (OCD) is a debilitating mental health disorder affecting 1−3% children and adolescents globally (Flament et al., 1988; Zohar et al., 1992; Valleni-Basile et al., 1994; Douglass et al., 1995; Stewart et al., 2004). Pediatric OCD is often accompanied by various comorbid conditions, including major depressive disorder, anxiety disorders, disruptive behavior disorders, and attention-deficit/hyperactivity disorder (Sharma et al., 2021). These comorbidities often exacerbate impairments in quality of life for affected children and adolescents (Geller et al., 1996, 2001, 2003; Masi et al., 2004, 2006; Sukhodolsky et al., 2005; Termine et al., 2006; Sharma et al., 2021).

Cognitive-behavioral therapy (CBT) with Exposure and Response Prevention (E/RP) is widely recognized as the first-line treatment for pediatric OCD (National Collaborating Centre for Mental Health [NCCMH], 2006). Previous research has consistently shown moderate to large effect sizes for CBT in treating OCD in children (McGuire et al., 2015; Skarphedinsson et al., 2015; Torp et al., 2015b). Moreover, CBT has demonstrated certain advantages over selective serotonin reuptake inhibitors (SSRIs) (Öst et al., 2016). Although limited, previous evidence suggests that CBT can reduce the impact of comorbid symptoms in pediatric OCD. For example, a meta-analysis (Sánchez-Meca et al., 2014) about the differential efficacy of CBT, pharmacological and combined treatment for pediatric OCD including 18 studies, concluded that all types of interventions were efficacious not only in reducing obsessive–compulsive symptoms, but also improved depression, anxiety, and other secondary responses, especially with CBT interventions.

Despite its promise, access to CBT still remains limited due to various barriers, including a lack of experienced therapists, long waiting lists, geographical limitations, and high costs (Franklin et al., 2013). Technological innovations present a potential solution to these challenges (Storch et al., 2011; Farrell et al., 2016; Lenhard et al., 2017). In this context, we developed an enhanced CBT (eCBT) package aiming to improve both access and treatment outcomes (Wolters et al., 2020). The primary studies explored, respectively, feasibility and accessibility (Babiano-Espinosa et al., 2021), and efficacy (Babiano-Espinosa et al., 2022) of eCBT, demonstrating significant reduction in OCD symptom severity [as measured by the Children’s Yale-Brown Obsessive-Compulsive Scale (CY-BOCS)], with 76% of participants classified as responders (CY-BOCS total score ≤15) and mean reduction of 63.8% on the CY-BOCS at post-treatment. The primary study (Babiano-Espinosa et al., 2022) also reported further decreases in CY-BOCS scores at 3 and 6-month follow-ups.

Besides the importance of primary outcomes, the limited attention to secondary outcomes such as comorbid symptoms, quality of life (QoL), and family accommodation constitutes a significant gap in the literature (Ginsburg et al., 2008; Lack et al., 2009; Weidle et al., 2014; Torp et al., 2015a). Addressing secondary outcomes is not merely an academic exercise, they are frequently associated with poorer primary treatment outcomes and are key indicators of treatment’s broader impact. For instance, family accommodation, which occurs within the context of family dynamics, can be highly resistant to change. Previous studies have indicated that children may be more reluctant to participate in treatment and refrain from confronting their obsessional fears, making symptom attenuation less likely (Ginsburg et al., 2008; Peris et al., 2008; Merlo et al., 2009; Garcia et al., 2010). Furthermore, early reductions in family accommodation have been shown to correlate with treatment effects at post-treatment (Piacentini et al., 2011; Peris et al., 2017) and during long-term follow-up (Piacentini et al., 2011). Moreover, comorbid symptoms are frequently associated with poorer primary treatment outcomes (Ginsburg et al., 2008; Torp et al., 2015a), and children and adolescents with OCD tended to experience increased comorbidity and reduced QoL compared to those without OCD (Lack et al., 2009; Weidle et al., 2014; Storch et al., 2018; Zaboski et al., 2019; Jensen et al., 2022).

The current study aims to explore the impact of eCBT on secondary outcomes, i.e., comorbid symptoms, QoL, and family accommodation in children and adolescents with OCD. We hypothesize that eCBT for OCD will reduce OCD-related comorbid symptoms and family accommodation, as well as increase quality of life.

## Materials and methods

### Study design

The open trial study took place at Child and Adolescent Mental Health Services (CAMHS) in St. Olav’s University Hospital, Trondheim, Norway (*n* = 23) and CAMHS in Hospital of Aalesund, Norway (*n* = 2) from January 2018 to February 2020. Assessments were made at baseline (week 0), post-treatment (week 14), 3-month follow-up, 6-month follow-up, and 12-month follow-up.

### Treatment and procedures

Enhanced cognitive-behavioral treatment (eCBT) is an innovative treatment package for children and adolescents with OCD. The treatment manual is derived from a NordLOTS manual (Jensen et al., 2022) and a Dutch treatment manual (Piacentini et al., 2007) for pediatric OCD. eCBT uniquely contains face-to-face sessions – covering psychoeducation, E/RP, cognitive interventions, and relapse prevention – with the flexibility of home-based videoconferencing sessions. The direct interaction in face-to-face sessions potentially strengthens the therapeutic alliance and provides comprehensive observational insights that webcam interactions may miss (Wolters et al., 2020). Videoconferencing, on the other hand, facilitates E/RP exercises in home environment or other natural settings. This approach aims to enhance the ecological validity of the treatment and encourage the application of CBT principles beyond the therapist’s office, directly into the settings where the problems naturally occur (Wolters et al., 2020). Compared to traditional CBT, eCBT offers more frequent therapist contact, overcoming geographical limitations. Additionally, eCBT incorporates an app to support psychoeducation and structured E/RP between sessions.

Enhanced cognitive-behavioral treatment (eCBT) covered a 14-weeks treatment period. The first part of the treatment (week 1−5) consists of weekly (45 min) face-to-face sessions, equivalent to traditional CBT. In the first week, psychoeducation videos and the eCBT concept (including the smartphone application system) are introduced to the patients and parents. From week 2, the E/RP exercises are modelled in the office followed by homework exercises based on an individualized OCD symptom inventory provided by children and parents. As soon as the child starts with E/RP homework (week 2), treatment is supplemented by a weekly videoconferencing (15 min) meeting, resulting in two appointments (one face-to-face session and one video conferencing session) with the therapists per week. During the additional webcam sessions, homework is reviewed and E/RP exercise at home or at another location can be performed. From week 6 onwards, the frequency of the face-to-face sessions is reduced to bi-weekly, while the frequency of webcam sessions (guided E/RP at home) is increased, resulting in one face-to-face session and two videoconferencing meetings in a two-week period. This default schedule provides the possibility to perform therapist assisted E/RP exercises in a natural environment. The frequency of sessions could be altered and personalized to patients’ needs but could not exceed the total number of sessions (a maximum of 10 face-to-face sessions and up to 12 shorter webcam sessions).

Treatment sessions are coordinated in a smartphone application (app) system. The app system consists of separate apps for children and parents, and a web-based application for therapists with a coordinating and monitoring function. All the apps are interconnected to synchronize the treatment outcome. The app provides information about OCD and CBT in the form of psychoeducation videos, supports, and structures E/RP at home, and closely monitors treatment progress. It can be used either in treatment sessions together with the therapists or independently at home. The app has been developed for the Android system, and children who had iPhone were given Android phones by the study staff.

### Participants

Participants were eligible for the study based on these criteria: (a) aged between 7−17 years; (b) a primary Diagnostic and Statistical Manual of Mental Disorders (DSM)-5 diagnosis of OCD^1^ ; (c) CY-BOCS score ≥16.

We excluded individuals who met any of the following conditions: (a) the presence of psychiatric comorbidity that has a higher treatment priority than OCD or rendering participation clinically inappropriate (examples include primary anorexia nervosa, depression with suicidality, and psychosis); (b) current engagement in psychological treatment specifically for OCD; (c) significant developmental delays; (d) inadequate proficiency in the Norwegian language.

Concurrent medications except for OCD were allowed during the study (Babiano-Espinosa et al., 2021). Among the participants, five were on medication: two on Methylphenidate for Attention deficit/hyperactivity disorder (ADHD), one on Guanfacine for tics and ADHD, one on Risperidone for eating disorder, and one on Aripiprazole for bipolar disorder. None were using SSRIs or other medications for anxiety or depression (Babiano-Espinosa et al., 2021).

### Measures

#### Comorbid symptoms and impairments

*Child Obsessive-Compulsive Impact Scale-Revised* (COIS-R) measures OCD-specific functional impairment of children and adolescents (Piacentini et al., 2007). There are separate child- and parent-rated questionnaires, each consisting of 33 items and a total score ranging from 0 to 99. A higher score indicates more severe functional impairment. The COIS-R demonstrated good internal consistency for both parent report (α = 0.83−0.91) and children report (α = 0.78−0.92) (Piacentini et al., 2007).

*Strengths and Difficulties Questionnaire* (SDQ) is a clinical tool to measure psychological adjustment in children and adolescents (Goodman, 1997). It is a broader measure of child’s mental health symptoms. The SDQ includes both a youth self-report and a parent-proxy version, each consisting of 25 items divided into five subscales. These subscales assess various aspects of a child’s emotional and behavioral functioning: emotional symptoms, conduct or behavior problems, hyperactivity-inattention, peer problems, and prosocial behavior. In our study, we use the Total Difficulties Score of SDQ (scoring from 0−40) to provide a measure of general psychological functioning from the perspectives of both the child and their parent. The Total Difficulties score includes the four problem-based subscales (emotional symptoms, conduct problems, hyperactivity-inattention, and peer problems). The Total Difficulties Score of the SDQ has shown satisfactory psychometric properties in children and adolescents (α = 0.74−0.80) as well as parents’ proxy samples (α = 0.76) (Van Roy et al., 2008).

*Screen for Child Anxiety-Related Emotional Disorders* (SCARED) is a 50-item child and parent self-support instrument (score 0−40) for child anxiety screening (Birmaher et al., 1997). A total score of ≥25 may indicate the presence of an anxiety disorder. Both the child and parent SCARED showed adequate internal consistency (α = 0.74−0.93) and good test-retest reliability (*r* = 0.70−0.90, intraclass correlation coefficient) (Birmaher et al., 1997).

*Mood and Feelings Questionnaire, short version* (MFQ) is a 13-item children and parent-rated measure assessing recent depressive symptomatology in children aged 6−17 years (Angold et al., 1995). Scores on the MFQ range between 0 to 26, with higher scores indicating greater depressive severity of a child. MFQ showed good internal consistency in both children (α = 0.76) and parent version (α = 0.87) (Sucupira et al., 2017).

#### Quality of life

*Questionnaire for Measuring Health-related Quality of Life in Children and Adolescents, revised version* (KINDL-R) is a well-established QoL questionnaire used in children and adolescents (Ravens-Sieberer et al., 2008). The present study employed both the self-reported questionnaire for children and adolescents and the parent proxy version. The KINDL-R questionnaire consists of 24 items which the participants were asked to rate on a five-point Likert scale (1 = *never*, 5 = *always*). The questionnaire is equally distributed into six subscales, representing the child’s well-being in physical, emotional, self-esteem, family, friends, and school over the past week. All subscale scores can be combined for a total QoL score, transformed to a scale ranging from 0−100. A higher score represents a better quality of life. In addition, the questionnaire provides an independent disorder-related subscale to produce information on the perceptions of OCD-related burden. Psychometric testing of the KINDL-R revealed good scale utilization (except for the family subscale showing ceiling effects of 17%) and sound scale fit (85%) (Bullinger et al., 2008). The internal consistencies for subscales ranged from α = 0.54 (friends and school) to α = 0.73 (family) and an α = 0.82 for the total score (Bullinger et al., 2008).

#### Family accommodation

*Family Accommodation Scale* (FAS) is a 12-item clinician-rated questionnaire with a total score ranging from 0−48 (Calvocoressi et al., 1999). It measures the extent to which the family members accommodate the child’s OCD symptoms as well as distress/impairment related to such accommodation. The FAS demonstrates satisfactory interrater reliability ranging from 0.72−1.0, as measured by intraclass correlation coefficients, and good internal consistency (α = 0.76) (Calvocoressi et al., 1999).

### Analysis

COIS-R, SDQ, SCARED, MFQ, and KINDL was filled in by 25 children and 25 parents. FAS was filled by 25 parents. Statistical analyses on secondary outcome variables at Baseline, Post CBT, 3-month follow-up (FU3), 6-month follow-up (FU6), and 12-month follow-up (FU12) were conducted using linear mixed-effected (LME) models (Supplementary Table 1). The fixed effect was time (Baseline, Post CBT, FU3, FU6, and FU12) and was treated as a categorical variable. Random effects included intercept and linear slope terms. Missing data were handled with maximum likelihood estimation as part of the LME. For each variable, mean value and standard deviation were reported with *p*-value and 95% confidence interval. To understand whether the change in secondary outcomes was related to the change of time, the relationship between baseline and follow-up treatment change in each variable score were calculated and reported as correlation coefficients (see Supplementary Table 1). Analyses were conducted in Statistical Analysis System (SAS) and Statistical Package for the Social Sciences (SPSS).

## Results

All 25 patients enrolled and completed eCBT treatment program (Table 1). Mean age of participants (*N* = 25) was 13.1 years (2.9) with slightly more girls (56%) than boys. Most of the participants (84%) lived with parents. Anxiety disorder (24%) was the most prevalent comorbid disorder among participants, followed by Tic disorder (16%) and autism spectrum disorder (ASD, 16%) (Table 1). Details regarding the administration and assessment of CY-BOCS and K-SADS PL have been described elsewhere (Wolters et al., 2020; Babiano-Espinosa et al., 2021).

**TABLE 1:** Baseline demographic and clinical characteristics of sample (*N* = 25).

Child age, in years, mean (SD)	13.1 (2.9)
**Gender, *N* (%)**	
Male	11 (44.0)
Female	14 (56.0)
**Parental status**	
Parents living together	21 (84.0)
Other	4 (16.0)
Age of OCD onset, in years, mean (SD)	9.7 (3.7)
**Baseline symptom severity (CY-BOCS), mean (SD)**	
Total score	25.8 (5.4)
Obsession score	13.1 (2.7)
Compulsion score	12.6 (3.1)
**Baseline comorbid diagnosis (K-SADS PL), *N* (%)**	
Any anxiety disorder	6 (24.0)
Any depressive disorder	1 (4.0)
ADHD	3 (12.0)
ODD/CD	0 (0)
Tic disorder	4 (16.0)
ASD	4 (16.0)
Encopresis	1 (4.0)
PTSD	2 (8.0)
Eating disorder	2 (8.0)
**Number of comorbid diagnoses, *N* (%)**	
None	13 (52.0)
1	8 (32.0)
2	2 (8.0)
≥3	2 (8.0)

*CY-BOCS*, Children’s Yale-Brown Obsessive-Compulsive Scale; *K-SADS PL, Kiddie* Schedule for Affective Disorders and Schizophrenia for School-Aged Children – Present and Lifetime Version; *ADHD*, Attention deficit/hyperactivity disorder; *ODD*, Oppositional defiant disorder; *OCD*, Obsessive–compulsive disorder; *ASD*, Autism spectrum disorder; *PTSD*, Post traumatic stress disorder.

### Secondary outcomes

#### Comorbid symptoms and impairments

In the child-reported version of COIS (COIS-C), the baseline score for functional impairment was significantly higher (*p* < 0.001) than the score at all other subsequent assessment points, including the post-treatment evaluation (Tables 2, 3). This suggests that functional impairment reduces following the treatment and that improvement is maintained through FU12. Childrens’ post-treatment scores were not significantly different from those at the FU-points, suggesting that functional impairment reduces following treatment, and that improvement is maintained through the follow up period. In the parent-reported version of COIS (COIS-P), the scores also significantly decreased at all assessment points. The most rapid decrease was observed at the FU3 (*p* < 0.001), with a more gradual but still significant decline seen at post treatment evaluation (*p* < 0.05).

**TABLE 2:** Reported outcomes across assessment points.

	Estimate	SE	95% CI	
			Lower bound	Upper bound
***COIS-C***				
Baseline	21.18	2.84	15.51	26.85
Post CBT	9.46	3.03	3.41	15.52
3M FU	6.88	3.39	0.10	13.66
6M FU	5.28	3.09	−0.90	11.46
12M FU	5.80	3.08	−0.37	11.97
***COIS-P***				
Baseline	27.27	4.11	19.06	35.49
Post CBT	17.23	4.49	8.25	26.21
3M FU	7.61	4.70	−1.79	17.00
6M FU	11.49	4.50	2.51	20.48
12M FU	13.49	4.16	5.18	21.81
***SDQ-C***				
Baseline	13.25	1.10	11.06	15.44
Post CBT	9.52	1.14	7.24	11.81
3M FU	10.81	1.28	8.27	13.36
6M FU	10.15	1.25	7.66	12.64
12M FU	9.94	1.16	7.62	12.25
***SDQ-P***				
Baseline	12.46	1.17	10.11	14.80
Post CBT	8.32	1.19	5.94	10.69
3M FU	7.46	1.33	4.80	10.11
6M FU	8.87	1.25	6.37	11.37
12M FU	9.30	1.19	6.93	11.68
***SCARED-C***				
Baseline	27.50	3.37	20.77	34.23
Post CBT	17.05	3.48	10.11	24.00
3M FU	18.48	3.68	11.13	25.82
6M FU	16.63	3.55	9.55	23.72
12M FU	18.20	3.48	11.26	25.15
***SCARED-P***				
Baseline	26.33	3.44	19.47	33.18
Post CBT	17.93	3.50	10.94	24.91
3M FU	14.70	3.78	7.15	22.24
6M FU	16.18	3.62	8.95	23.41
12M FU	17.56	3.50	10.58	24.54
***MFQ-C***				
Baseline	7.22	1.12	4.98	9.46
Post CBT	3.99	1.18	1.63	6.35
3M FU	4.67	1.29	2.09	7.26
6M FU	5.95	1.22	3.51	8.39
12M FU	5.65	1.20	3.26	8.05
***MFQ-P***				
Baseline	7.17	1.08	5.02	9.32
Post CBT	4.48	1.09	2.30	6.67
3M FU	3.64	1.24	1.17	6.12
6M FU	3.68	1.16	1.37	5.99
12M FU				
***KINDL-C***				
Baseline	61.75	2.93	55.90	67.61
Post CBT	69.91	3.02	63.88	75.94
3M FU	68.29	3.29	61.72	74.86
6M FU	69.15	3.11	62.94	75.37
12M FU	69.86	3.02	63.84	75.88
***KINDL-P***				
Baseline	58.70	2.93	52.85	64.55
Post CBT	69.24	2.98	63.29	75.20
3M FU	70.77	3.29	64.20	77.33
6M FU	69.74	3.09	63.57	75.91
12M FU	67.94	2.93	62.09	73.80
***FAS***				
Baseline	19.01	1.86	15.30	22.73
Post CBT	5.77	1.94	1.91	9.63
3M FU	2.80	2.02	−1.24	6.83
6M FU	4.14	2.07	0.01	8.27
12M FU	4.94	1.93	1.09	8.79

Estimations of outcome measures’ scores based on linear mixed-effects model. Post CBT was measured as week 14. *FU*3 3 months follow-up, *FU6* 6 months follow-up, *FU12* 12 months follow-up, *SE* standard error. *COIS-R*, Child Obsessive-Compulsive Impact Scale-Revised; *SDQ*, Strengths and Difficulties Questionnaire; *SCARED*, Screen for Child Anxiety-Related Emotional Disorders; MF, Mood and Feelings Questionnaire short version; *KINDL-R*, Questionnaire for Measuring Health-related Quality of Life in Children and Adolescents, revised version; *FAS*, Family Accommodation Scale; -*C*, Child report; -*P*, Parent report.

**TABLE 3:** Comparisons of outcome changes across follow-up assessment points (*P* < *0.05*).

Estimate (SE)COIS-C	COIS-P	SDQ-C	SDQ-P	SCARED-C	SCARED-P	MFQ-C	MFQ-P	KINDL-C	KINDL-P	FAS
Baseline vs. Post 11.72 (3.17)***	10.04 (4.62)*	3.73 (1.10)**	4.14 (1.19)***	10.45 (2.52)***	8.40 (2.82)**	3.23 (1.11)**	2.69 (1.18)*	−8.15 (2.87)**	−10.54 (3.07)**	13.25 (2.31)***
Baseline vs. FU314.30 (3.53)***	19.67 (4.87)***	2.43 (1.24)	5.00 (1.33)***	9.02 (2.80)**	11.63 (3.18)***	2.55 (1.23)*	3.53 (1.32)**	−6.53 (3.17)*	−12.06 (3.40)***	16.22 (2.39)***
Baseline vs. FU615.90 (3.21)***	15.78 (4.62)**	3.10 (1.21)*	3.59 (1.25)**	10.87 (2.62)***	10.15 (2.96)***	1.27 (1.15)	3.49 (1.24)**	−7.40 (2.97)*	−11.04 (3.17)***	14.88 (2.43)***
Baseline vs. FU1215.38 (3.18)***	13.78 (4.34)**	3.31 (1.11)**	3.16 (1.19)**	9.30 (2.50)***	8.76 (2.80)**	1.57 (1.12)	1.22 (1.18)	−8.11 (2.82)**	−9.24 (3.00)**	14.08 (2.34)***
Post vs. FU32.58 (3.63)	9.62 (5.10)	−1.29 (1.27)	0.86 (1.33)	−1.42 (2.87)	3.23 (3.19)	−0.68 (1.26)	0.84 (1.32)	1.62 (3.16)	−1.52 (3.34)	2.97(2.44)
Post vs. FU64.18 (3.40)	5.74 (4.93)	−0.63 (1.26)	−0.55 (1.27)	0.42 (2.76)	1.75 (3.04)	−1.96 (1.20)	0.80 (1.26)	0.76 (3.01)	−0.50 (3.19)	1.63 (2.48)
Post vs. FU123.67 (3.41)	3.74 (4.76)	−0.41 (1.16)	−0.99 (1.21)	−1.15 (2.66)	0.37 (2.91)	−1.66 (1.20)	−1.47 (1.21)	0.05 (2.94)	1.30 (3.08)	0.83 (2.40)
FU3 vs. FU61.60 (3.66)	−3.89 (5.06)	0.66 (1.34)	−1.41 (1.37)	1.85 (2.93)	−1.48 (3.29)	−1.28 (1.29)	−0.04 (1.37)	−0.86 (3.23)	1.03 (3.42)	−1.34 (2.54)
FU3 vs. FU121.08 (3.75)	−5.89 (4.93)	0.88 (1.30)	−1.84 (1.35)	0.28 (2.94)	−2.86 (3.24)	−0.98 (1.32)	−2.31 (1.34)	−1.57 (3.24)	2.82 (3.37)	−2.14 (2.47)
FU6 vs. FU12−0.52 (3.44)	−2.00 (4.73)	0.22 (1.27)	−0.43 (1.27)	−1.57 (2.75)	−1.38 (3.04)	0.30 (1.23)	−2.26 (1.26)	−0.71 (3.04)	1.80 (3.16)	−0.80 (2.51)

*FU*3 3 months follow-up, *FU6* 6 months follow-up, *FU12* 12 months follow-up, *SE* standard error. *COIS-R*, Child Obsessive-Compulsive Impact Scale-Revised; *SDQ*, Strengths and Difficulties Questionnaire; *SCARED*, Screen for Child Anxiety-Related Emotional Disorders; *MFQ*, Mood and Feelings Questionnaire, short version; *KINDL-R*, Questionnaire for Measuring Health-related Quality of Life in Children and Adolescents, revised version; *FAS*, Family Accommodation Scale; -*C*, Child report; -*P*, Parent report.

*P < 0.05,

**P < 0.01,

***P < 0.001.

Child-reported SDQ (SDQ-C) scores improved from baseline to post-treatment (mean reduce = 3.73, SE = 1.10, *p* < 0.01). No significant change was observed between baseline and FU3 (*p* = 0.054) or in subsequent follow-ups, suggesting that improvement in child-reported emotional and behavioral functioning was maintained over time. In the parent reported version, a significant improvement was observed at post-treatment (*p* < 0.001) and at FU3 (*p* < 0.001). In addition, further improvements were observed at FU6 and FU12 (*p* < 0.01).

Compared to baseline, significant reductions were observed in the child-reported SCARED scores post-treatment (mean reduce = 10.45, SE = 1.10, *p* < 0.001) and at all subsequent FUs (FU3: mean reduce = 9.02, SE = 2.80, *p* < 0.01; FU6: mean reduce = 10.87, SE = 2.62, *p* < 0.001; FU12: mean reduce = 9.30, SE = 2.50, *p* < 0.001). Similar significant improvements were found in SCARED-P, also these were maintained throughout the FU period.

In both MFQ-C and MFQ-P, the baseline score was significantly higher than post-treatment and any subsequent assessment point (Tables 2, 3). This indicates that depressive symptoms reduced following treatment and this reduction was maintained up to the FU12.

#### Quality of life

Both child- and parent-rated KINDL-R scores have improved significantly from baseline to post-treatment, and from baseline to the FU3 (*p* < 0.001) (Table 3). Specifically, there was a mean increase of 3.73 points (SE = 1.57) from baseline to post treatment, and 6.93 points (SE = 2.92) from baseline to FU3 for children. For parents, the corresponding increase was 6.57 points (SE = 1.67) from baseline to post and a 12.20-point increase (SE = 3.09) from baseline to FU3.

#### Family accommodation

Family Accommodation Scale (FAS) scores showed a significant decrease at post-treatment compared to baseline (p < 0.001) (Table 3). Mean decrease was 8.65 points (SE = 1.21) from baseline to post-treatment, and 16.06 points (SE = 2.26) compared from baseline to FU3 for children’s scores.

## Discussion

The present study investigated the impact of eCBT for pediatric OCD on comorbid symptoms, OCD-related functional impairment, quality of life, and family accommodations. Overall, we observed a statistically significant reduction in all secondary outcomes from baseline to post-treatment, which was maintained through the 12-month follow-up period. These results suggest that the participants experienced significant improvements in daily functioning across various domains, including home life, schooling, and social interactions. Our findings conveyed a positive message, suggesting that the treatment effects of pediatric OCD may extend beyond merely reducing OCD symptoms. This is in line with an early study by (Conelea et al., 2017), which observed improvement in non-OCD anxiety, inattention, hyperactivity, and overall quality of life following CBT for pediatric OCD. Furthermore, the decreased OCD-related functional impairment evidenced by our results resonate with previous research (Storch et al., 2011; Bonnert et al., 2014), reinforcing the idea that effects of treatment are broader than solely targeting OCD symptoms.

We observed a reduction in general psychological adjustment in children and adolescents, as reflected in the total score of the SDQ (both parents and the young people), from baseline to post-treatment and at three-month follow-up. This finding indicates that eCBT demonstrated an overall positive impact on youth with OCD in various domains – spanning child’s overall emotional well-being, behavior, attention, peer relations, and prosocial behavior – as rated by both children and parents. Particularly, the co-occurring emotional problems, such as depressive symptoms, witnessed a significant decline, as measured by the MFQ scores (both parents and the young people) from baseline to post-treatment and sustained through the 12-month follow up. This outcome supports our hypothesis that effectively treating OCD symptoms may enhance overall mood, particularly considering that many children with OCD often experience depression due to the restrictive nature of their disorder in social life (Griffiths et al., 2012). The reduction of OCD likely lessens this burden. During eCBT, pediatric patients learned techniques to manage obsessions and other intrusive thoughts that can also contribute to depression and anxiety. These techniques can also be beneficial for treating non-OCD anxiety symptoms, thereby improving comorbid symptoms. Our findings resonate with those from a large previous meta-analysis conducted by Sánchez-Meca et al. (2014), which reported improvements in depression, anxiety, and other secondary responses following treatment, irrespective of the modality (CBT, medication, or their combination), with CBT showing particular effectiveness.

Another noteworthy improvement in emotional well-being in our study was the significant reduction in overall anxiety symptoms, as evidenced by the score of SCARED (both parents and the young people), from baseline to post-treatment, and consistently maintained at the three-month follow-up. These reductions encompass a range of anxiety symptoms, including generalized anxiety, panic symptoms, separation anxiety symptoms, social anxiety symptoms and anxiety related to school. Given the high prevalence of OCD and co-occurring anxiety symptoms (Banneyer et al., 2018), the observed reduction in anxiety symptom levels following CBT in pediatric OCD cases holds clinical importance. Our results mirror those of a recent study by Babiano-Espinosa et al. (2022), which underscored the effectiveness of eCBT in reducing anxiety symptoms and stress. Furthermore, our findings are in line with several previous research in this domain. For example, a study by (Cancilliere et al., 2018) found that children aged 5−8 years (*n* = 127) who underwent family-based CBT for OCD exhibited significantly reduced comorbid anxiety symptoms than those receiving family-based Relaxation Therapy. Similarly, Conelea et al. (2017) reported a reduction in comorbid anxiety and inattention symptoms among OCD patients treated with CBT in combination with medication.

Aligned with our hypothesis, we observed significant reductions in OCD-specific functional impairment, as measured by COIS scores (from both parents and the young people), at post-treatment. The gains were maintained through the 12-month follow-up. This pattern of improvements mirrors findings from the Nordic Long-term OCD treatment Study (NordLOTS) by Højgaard et al. (2017), where both patient- and parent-rated functioning in pediatric patients showed maintained enhancements one year post-CBT. Similarly, our results resonate with the study by Bolton et al. (2011), which also reported a significant reduction in the impact of pediatric OCD on level of functioning. Furthermore, we observed a significant overall decrease in family accommodation over time, which is in line with the trends observed in previous studies (Piacentini et al., 2011; Thompson-Hollands et al., 2015; Peris et al., 2017). This outcome holds clinical importance in treating pediatric OCD, as evidence reveals that higher levels of family accommodation (e.g., specific contamination/cleaning symptoms and behavioral manifestations) are often linked with increased severity of OCD symptoms (Skarphedinsson et al., 2023).

Both children and parents reported an improvement in quality of life (QoL) from baseline to post-treatment, aligning with findings from previous studies (Weidle et al., 2015; Storch et al., 2018). Interestingly, we observed further improvement from baseline to the three-month follow-up, which persisted throughout the follow-up period. This additional improvement during the first 3°months after treatment may be indicative of a phase where skills and gains acquired during therapy are integrated into daily life, leading to a continued improvement in QoL beyond mere symptom reduction. This observation is consistent with the understanding from earlier research by Weidle, 2015, which documented that alleviating OCD symptoms has a positive impact on children’s overall quality of life and well-being.

### Strengths and limitations

The strength of our study is a comprehensive evaluation of outcome of OCD-related impairments beyond OCD symptom reduction after treatment in pediatric OCD employing reliable instruments and a multi-perspective approach, representing both children and parents. Another strength is the assessment of comorbid symptoms, OCD-related impairments quality of life, and family accommodation with follow-up points over a span of 12 months.

However, the main limitation for interpreting the results from our study is relatively small sample size, due to the pilot character of the intervention. This limits generalizability of the findings. However, our study sample comprises patients exhibiting moderate to severe OCD and a high prevalence of comorbid symptoms which seems to be demographic representative for patients usually referred for OCD treatment to child psychiatric services. In addition, we acknowledge the potential for type I error due to not correcting for multiple comparisons. Our methodology, using linear-mixed effects models, mitigates this risk by adjusting for correlations within repeated measures. Nonetheless, the exploratory nature of our study and the conventions of related research allow for this approach, though it may constrain the interpretability of our results. In addition, as an open-label trial study lacking a control group, the potential influence of other factors such attention, spontaneous remission, or regression to the mean, cannot be ruled out. Another limitation is that eCBT was facilitated through an app designed for the Android platform only. Hence, children with iPhones and no access to an Android device had to borrow one for the study’s duration, including the need to manage and maintain the function of two devices, potentially influencing their motivation and engagement for treatment.

## Conclusion

Enhanced cognitive behavioral therapy (eCBT) for pediatric OCD decreased OCD-associated comorbid anxiety- and depressive symptoms, impairments of daily life functioning and improved quality of life beyond OCD symptom reduction. These improvements were sustained over a period of time.

## Data availability statement

The raw data supporting the conclusions of this article will be made available by the authors, without undue reservation.

## Ethics statement

The studies involving humans were approved by the Regional Committee for Medical and Health Research Ethics (No 2016/716/REK Nord) and registered with the ISRCTN (https://www.isrctn.com/) registry (trial ID: ISRCTN37530113). The studies were conducted in accordance with the local legislation and institutional requirements. Written informed consent for participation in this study was provided by the participants’ legal guardians/next of kin.

## Author contributions

BoW: Conceptualization, Data curation, Investigation, Methodology, Software, Visualization, Writing – original draft, Writing – review & editing. GS: Software, Supervision, Validation, Writing – review & editing. BeW: Resources, Writing – review & editing. LB-E: Writing – review & editing. LW: Writing – review & editing. JA: Writing – review & editing. NS: Funding acquisition, Project administration, Resources, Writing – review & editing.

## Footnotes

1 The OCD clinical diagnoses were established based on CY-BOCS scores and ICD-10/DSM-5 criteria.
