# Supplementary material for: Secondary outcomes of enhanced cognitive behavioral therapy (eCBT) for children and adolescents with obsessive-compulsive disorder
Source: Front Hum Neurosci. 2024 Jan 8;17:1330435. doi: 10.3389/fnhum.2023.1330435 (PMC10800953; doi:10.3389/fnhum.2023.1330435)
Supplement: Supplementary file 1 [file Table_1.docx]

# Supplementary Material

Table S1. Parameter estimates for fitting the linear mixed effects models.

| ***Child rated*** |  |  |  |  |  |
| --- | --- | --- | --- | --- | --- |
| Composite model, estimate (SE) | COIS | SDQ | SCARED | MFQ | KINDL-R |
| Intercept | 20.25 (2.73)*** | 12.63 (1.07)*** | 26.18 (3.33)*** | 6.68 (1.10)*** | 63.04 (2.84)*** |
| Time1 ^1^ | -0.59 (0.12)*** | -0.11 (0.04)* | -0.40 (0.10)*** | -0.09 (0.04)* | 0.27 (0.11)* |
| Time2 ^2^ | 0.6097 (0.19)*** | 0.12 (0.07) | 0.46 (0.15)** | 0.13 (0.67) | -0.27 (0.17) |
| Variance components |  |  |  |  |  |
| Level-1-within person | 99.75 (17.69)*** | 13.97 (2.38)*** | 69.94 (11.85)*** | 13.61 (2.32)*** | 83.53 (14.29)*** |
| Level-2-in initial status | 88.20 (35.43)* | 15.94 (5.74)** | 212.74 (67.62)*** | 17.53 (6.22)** | 115.66 (40.12)** |
| ***Parent rated*** |  |  |  |  |  |
| Composite model, estimate (SE) | COIS | SDQ | SCARED | MFQ | KINDL-R |
| Intercept | 27.27 (3.98)*** | 11.98 (1.14)*** | 25.69 (3.36)*** | 7.00 (1.03)*** | 59.91 (2.82)*** |
| Time1 | -0.72 (0.17)*** | -0.18 (0.05)*** | -0.45 (0.11)*** | -0.15 (0.05)** | 0.47 (0.12)*** |
| Time2 | 0.86 (0.25)** | 0.24 (0.07)*** | 0.54 (0.16)** | 0.22 (0.07)** | -0.58 (0.18)** |
| Variance components |  |  |  |  |  |
| Level-1-within person | 188.94 (22.01)*** | 14.89 (2.52)*** | 82.22 (13.83)*** | 14.37 (2.44)*** | 92.08 (15.75)*** |
| Level-2-in initial status | 195.09 (72.84)** | 17.08 (6.19)** | 202.75 (65.64)** | 11.70 (2.44)** | 100.18 (36.38)** |

1 time coefficient before the change in slope

2 time coefficient after the change in slope

* *P* < .05, ** *P* <.01, *** *P* <.001.
